# Influences of Recreational Tennis-Playing Exercise Time on Cardiometabolic Health Parameters in Healthy Elderly: The ExAMIN AGE Study

**DOI:** 10.3390/ijerph18031255

**Published:** 2021-01-30

**Authors:** Hsiao-Han Chao, Yi-Hung Liao, Chun-Chung Chou

**Affiliations:** 1 Department of Athletics, National Taiwan University, Taipei 10617, Taiwan; nikkichao@ntu.edu.tw; 2 Department of Exercise and Health Science, National Taipei University of Nursing and Health Sciences, Taipei 11219, Taiwan; yihungliao.henry@gmail.com; 3 Physical Education Office, National Taipei University of Technology, Taipei 10608, Taiwan

**Keywords:** arterial stiffness, insulin resistance, racquet sports, physical activity, arterial compliance

## Abstract

Background: Aging and chronic degeneration are the primary threats to cardiometabolic health in elderly populations. Regular appropriate exercise would benefit the advanced aging population. Purpose: This study investigates whether the degree of weekly tennis participation exhibits differences in primary cardiometabolic parameters, including arterial stiffness, inflammation, and metabolic biomarkers in elderly tennis players. Methods: One hundred thirty-five long-term participants in elder tennis (>50 years old) were initially screened. Twenty-six eligible and voluntary subjects were divided into high tennis time group (HT) (14 ± 1.3 h/week) and low tennis time group (LT) (4.5 ± 0.7 h/week) by stratification analysis based on the amount of tennis playing activity time. The brachial-ankle pulse wave velocity (baPWV), blood pressure, ankle-brachial index (ABI), blood metabolic biomarkers, and insulin resistance were measured to compare the difference between HT and LT groups. Results: The baPWV was significantly lower in the HT group than that in the LT group (1283.92 ± 37.01 vs. 1403.69 ± 53.71 cm/s, *p* < 0.05). We also found that the HT insulin-resistant homeostasis model assessment (HOMA-IR) was significantly lower than that of LT (1.41 ± 0.11 vs. 2.27 ± 0.48 μIU/mL, *p* < 0.05). However, the blood lipid biomarkers (glucose, cholesterol, high-density lipoprotein cholesterol, low-density lipoprotein cholesterol, triglyceride) were not statistical different between HT and LT groups (*p* > 0.05). Conclusion: We demonstrated that under the condition of similar daily physical activity level, elderly with a higher time of tennis-playing (HT group) exhibited relatively lower arterial stiffness (lower PWV) and lower insulin resistance compared to those with lower time tennis-playing (LT).

## 1. Introduction

Lack of physical activity is the main risk factor for metabolic disorder and subsequent cardiovascular disease pathogenesis (CVD). It is well known that regular physical activity and exercise, particularly endurance exercise, were proven to enhance cardiovascular functions, insulin sensitivity, thereby decreasing the prevalence of cardiovascular diseases and the mortality from related complications during advancing aging [1,2,3]. Regular exercise training has clear protective benefits on enhancing overall glycemic control capacity, promoting insulin sensitivity, and improving lipid metabolism [4,5]. Recent evidence indicates that high-intensity interval training (HIIT) has also been suggested as another effective training modality for the prevention of CVD, enhancement of cardiorespiratory fitness, and improvement in cardiovascular risk profile in older adults [6,7].

Tennis is a non-contact and popular leisure exercise that is suitable for elderly populations. The features of tennis include long-term endurance activity for aerobic fitness advantage and consist of HIIT, power, and impact characteristics, that will have a positive benefit on cardiovascular function [8], aerobic capacity, and muscle mass [9,10]. Although the characteristics of tennis matches varied among different player styles and court surfaces, the previous research indicated that the intensity of tennis could be classified as moderately vigorous [9,11,12]. A recent epidemiological study showed that participation in racquet sports displayed a significant risk reduction in all-cause mortality (↓47%) and CVD mortality (↓59%) [13]. Another prospective cohort study revealed decreased CVD risk among tennis participants [14]. Moreover, the number of people engaged in high-intensity exercise may exhibit varying degrees in exercise-induced health benefits on cardiovascular health. Previous studies showed an association between more regular high-intensity exercise and lower levels of arteriosclerosis [15,16]. Moreover, HIIT seems to be an effective exercise model for improving metabolic health and insulin sensitivity [17,18]. A previous study suggested that HIIT can improve aerobic fitness and insulin resistance in sedentary older adults [19].

Arterial abnormality is one of the primary contributors to future CVD [20], and having a high level of atherosclerosis has been suggested to be the earliest symptoms of CVD pathogenesis [21]. The position statement of the American College of Sports Medicine (ACSM) has clearly stated that regular exercise participation is an effective non-pharmacological strategy for cardiovascular and metabolic health [22]. In the aspects of cardiovascular health, exercise interventions have been shown to improve vascular function, thereby reducing the incidence of CVD and mortality [1,2,3]. On the other hand, the underlying mechanisms by which exercise training improves metabolic health include the ameliorative changes in blood lipid profiles (e.g., triglycerides, total cholesterol, LDL, etc.), peripheral insulin sensitivity, and systemic glycemic control [4,5]. Furthermore, insulin resistance has been well recognized as one of the common origins of the development of abnormal blood pressure regulation and negative changes in arterial function/structure (increased arterial compliance) [23,24]. Even though few investigations revealed the point about tennis on cardiovascular function, however, the relationship between the tennis exercise amount and cardiovascular function, metabolic biomarkers, and insulin sensitivity among tennis elderly populations is still unknown.

Although participation in tennis training may produce certain degrees of health-related benefits, little is still known about whether the time of tennis participation elicits different cardiovascular and metabolic health biomarker benefits in elderly populations. In this aspect, this information would be critical to health care professionals in appropriately prescribing exercise training for this special population. We hypothesized that populations with higher tennis participation should associate with better in arterial stiffness and blood lipids biomarkers. This study investigates whether the degree of weekly tennis participation exhibits differences in primary cardiometabolic parameters, including arterial stiffness, inflammation, and metabolic biomarkers in elderly recreational tennis players. The results obtained in this study can provide evidence on cardiovascular health of the middle-aged and the elderly population through performing racquet sports, thus our findings raise the proposed feasibility of constructing more appropriate tennis or racquet sport facilities/environments in promoting public health in the populations with advancing aging.

## 2. Materials and Methods

### 2.1. Participants and Ethical Statement

**Figure 1:**
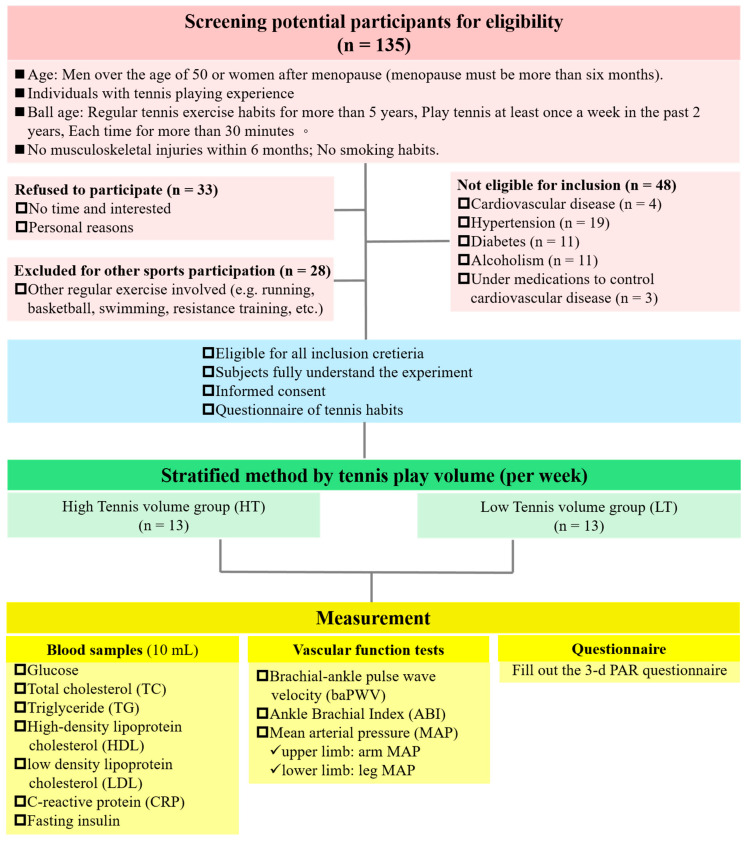
Flow chart of the study participant’s screening and recruitment over the study period.

One hundred thirty-five elder individuals (>50 years old) were initially screened for confirming whether they were eligible for this study. Twenty-six eligible voluntarily participants (age range: 53–70 years old; male: 15; female: 11) completed all tests, questionnaires, and blood sample collection. None of the participants smoked or were diagnosed as having cardiovascular diseases, hypertension, hyperlipidemia, diabetes mellitus, chronic renal failure, etc. To determine whether the differences in results were due to different levels of participation in tennis, those individuals with a history of regularly engaging in other types of sports were excluded from this study. The female participants were all at the postmenstrual phase and free of taking hormone replacement therapy for at least one year before the investigation. The purpose, experimental procedures, and potential risks of this study were clearly explained to each participant, and written informed consent was provided and signed by the eligible participants before the study initiation. The experimental protocol was approved by the Institute Review Board (IRB) of the Research Ethics Committee of Taipei Medical University (TMU-JIRB, N201703052). Moreover, the study protocol was performed according to the guidelines from the last version of the Declaration of Helsinki. The flow of study participants through the three screening and recruitment steps is shown in Figure 1.

### 2.2. Study Design

All eligible and voluntary subjects (*n* = 26) presented to the laboratory at 08:00 AM in fasting condition on the test day. After 10 min of rest, a vein blood sample was collected. The subjects were then asked to lie down quietly for 20 min for vascular function tests (pulse wave velocity, blood pressure, and ankle-brachial index). After vascular function testing, all subjects were requested to fill in physical activity logs and weekly tennis-playing records. The stratification analysis used in this study was mainly based on the median value (there were twenty-six participants joined this study, thus we take the rank #13 and #14 participants’ tennis playing time to calculate the median; ranking #13 = 10.0 h/week& ranking #14 = 8.25 h/week; median = 9.125 h/week) of the amount of tennis playing activity time recorded using physical activity questionnaire from all the participants. The tennis play time per week was used as the basis for the stratified analysis method in this study. The 26 voluntary subjects were then divided into a high tennis time group (HT) (14 ± 1.3 h/week) and a low tennis time group (LT) (4.5 ± 0.7 h/week).

### 2.3. Physical Activity and Exercise Intensity/Duration Analyses

All participants were required to record their regular physical activity using a three-day physical activity log (3-d PAL). This log included records forms for two weekdays and one weekend day [25]. In the 3-d PAL, the participants filled in the numbers from 1 to 9 according to the physical activity intensity. The higher the number, the higher the physical activity intensity. When the recorded number is 8 or 9 (1.5–2.0 kcal/kg/15 min), it means high-intensity physical activity. When the recorded number is 4 to 7 (0.69–1.4 kcal/kg/15 min), it means low to moderate physical exercise intensity.

### 2.4. The Calculation Method for Tennis Playing Time

The time engaged in tennis per week was obtained from the tennis habits investigation. The tennis-playing time per week was calculated as follows: Tennis-playing time per week (h/week) = frequency (day/week) × time (h/day).

### 2.5. Brachial-Ankle Pulse Wave Velocity (baPWV) and Other Hemodynamics Measurements

An automatic waveform analyzer (VP-1000, Omron Healthcare Co Ltd., Kyoto, Japan) was used to measure baPWV, blood pressure, and ABI simultaneously. Briefly, the subject after resting for at least 10 min in a supine position, the bilateral brachial arteries and tibial arteries were wrapped with four oscillometric cuffs connected to a plethysmographic sensor for baPWV measurement. In this study, the cuffs were confirmed to be tied to the arm (left and right upper arm brachial artery) and ankle (left and right lower limb ankle artery) during the first baPWV measurement. The subjects were measured twice in a continuous manner with a 1-min interval while lying. The pulse wave velocity from the right (left) arm to the right (left) ankle was automatically calculated as the distance/transit time between the right (left) arm and the right (left) ankle.

According to the regression equation, the assumed pulse travel distance between the arm and the ankle was automatically calculated: distance = 0.5934 × height + 14.401 [26]. The ABI index was calculated: right (left) lower limb systolic blood pressure/right (left) upper limb systolic blood pressure. The average baPWV and ABI on the left and right were used for analysis. During the test, heart rate and blood pressure from the upper and lower limbs, including systolic blood pressure (SBP), diastolic blood pressure (DBP), and mean arterial pressure (MAP), were recorded. All tests were measured twice, and the average of two measurements was used for results calculation.

### 2.6. Blood Metabolic Health Biomarkers Analyses

In the blood sample analyses, we collected whole blood samples in red-capped tubes containing coagulation activator with gel separator or anticoagulant reagent (ethylenediaminetetraacetic acid, EDTA). After blood collection, blood samples were left at room temperature for 30 min and then centrifuged at 3000× *g* for 10 min (4 °C) to separate the blood cells from the serum. Serum from each blood sample was used to determine circulating levels of metabolic biochemical parameters (glucose and lipid profiles), insulin, and CRP. Fasting venous blood samples (10 mL) was taken by a certified medical technologist or registered nurse for blood glucose, total cholesterol (CHOL), high-density lipoprotein cholesterol (HDL), low-density lipoprotein cholesterol (LDL), triglyceride (TG), C-reactive protein (CRP) and insulin analyses. The CHOL, LDL, HDL, and TG were measured using UniCel DxC-800 Synchron Clinical Systems (Beckman Coulter, Inc., Brea, CA, USA). The CRP and insulin concentration measurements were performed using the enzyme-linked immunosorbent assay method (ELISA) with commercial ELISA kits (CRP No 969,620 on a Beckman DxC800 analyzer, Beckman Brae, CA, USA; insulin NO 10111301, Mercodia AB, Uppsala, Sweden) used to determine the levels following the manufacturers’ instructions. In all measurements performed the coefficients of inter-and intra-assay were less than 10%.

### 2.7. Homeostasis Model Assessment of Insulin Resistance (HOMA-IR Index)

An indirect index, the homeostatic model assessment of insulin resistance index (homeostasis model assessment of insulin resistance, HOMA-IR index), was used for the insulin resistance (IR) assessment [27]. HOMA-IR was calculated using fasting glucose and insulin levels using the following formula: HOMA-IR = fasting blood glucose (mmol/L) × fasting insulin (μU/mL)/22.5.

### 2.8. Statistical Analyses

Statistical analyses were performed using SPSS 20.0 for Windows. The effect size (ES) for HT and LT was calculated using Cohen’s ∆ (d). The differences in all parameters between HT and LT groups were compared using the independent sample t-test. All data are expressed as mean ± standard error of the mean (Mean ± S.E.), and the alpha values of statistically significant for all comparisons are set to 0.05 (*p* < 0.05).

## 3. Results

### 3.1. Participant’s Anthropometry

Twenty-six voluntary and qualified subjects of a total of 135 people (19.3%) were recruited and completed all tests. In all subjects, 57.7% were men and 42.3% were women. There were no differences in anthropometry measurements (height, weight, BMI, age, and years of tennis-playing) between HT and LT groups (Table 1).

### 3.2. Amount of Tennis Playing and Physical Activity

**Figure 2:**
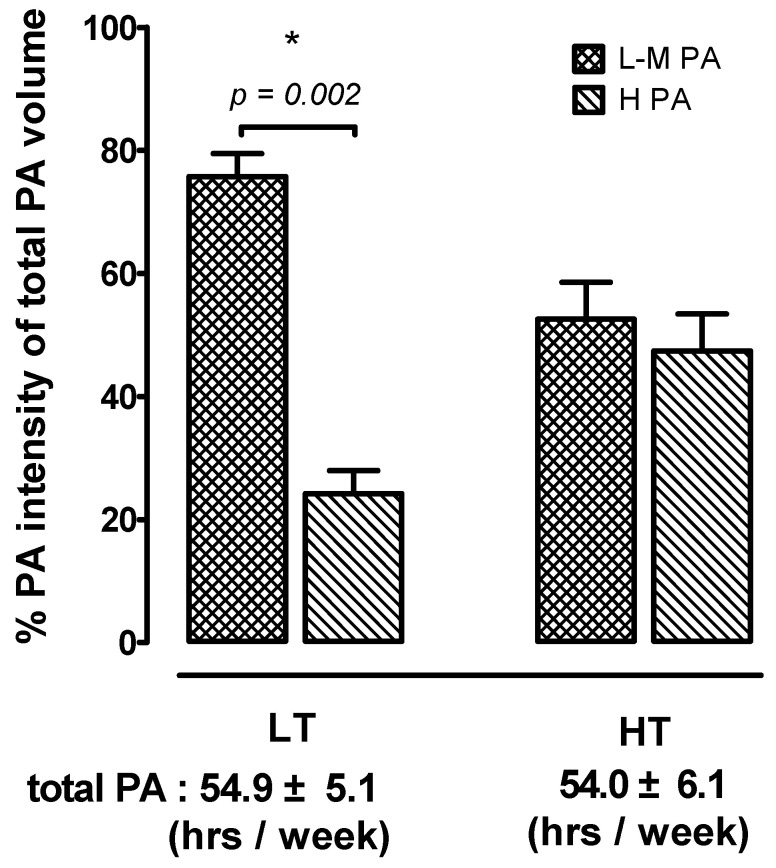
Differences in physical activity time per week in LT and HT. Data are expressed as Mean ± S.E.M. * denotes a significant difference between L-M PA and H PA (*p* < 0.05). Low tennis time group (LT), high tennis time group (HT), physical activity (PA), low to moderate physical activity (L-M PA), high physical activity (H PA).

According to the independent sample t-test, the frequency of play per week, hours of play per day, a total time of play per week, and high-intensity exercise were all significantly higher in the HT groups in comparison with the LT group (Table 1). There was no difference in the physical activity time per week between the two groups, however, the percentage of low to moderate physical activity (L-M PA) was significantly higher than the percentage of high physical activity (H PA) in the LT group (*p* < 0.05) (Figure 2).

### 3.3. Differences in Blood Metabolic Health-Related Biomarkers

According to the independent sample *t*-test, the HOMA-IR of HT was significantly lower than that of LT (*p* < 0.05). Nonetheless, the glucose, CHO, TG, HDL, LDL, and CRP were no statistical difference between HT and LT groups (*p* > 0.05) (Table 2).

### 3.4. Differences in baPWV to Varied Tennis Time

**Figure 3:**
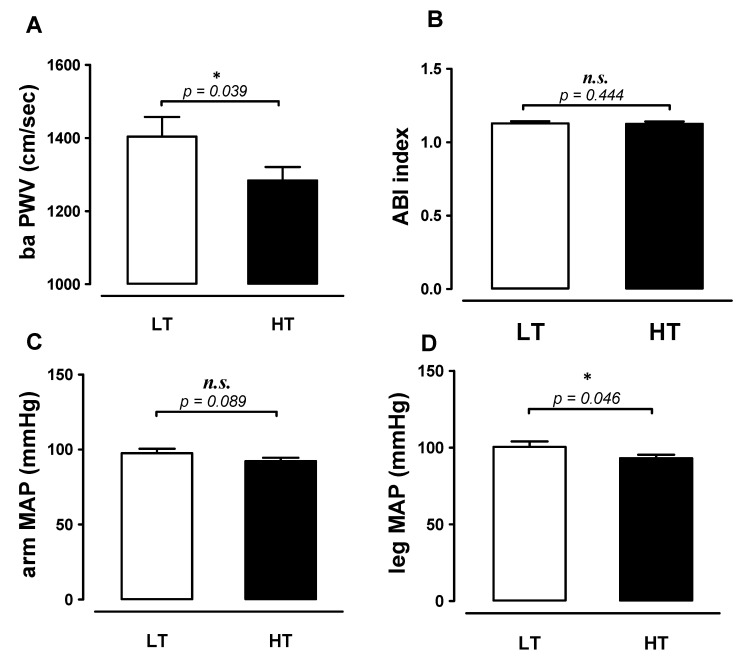
Differences in baPWV, ABI index, and blood pressure in LT and HT. (**A**) baPWV, (**B**) ABI index, (**C**) arm MAP and (**D**) leg MAP were measured in HT and LT groups (LT, open bar; HT, black bar). Data are expressed as Mean ± S.E.M. * denotes significant difference between two groups (*p* < 0.05). Low tennis time group (LT), high tennis time group (HT), ankle-brachial index (ABI index), mean arterial blood pressure (MAP).

According to the independent sample t-test, the levels of baPWV (1283.92 ± 37.01 vs. 1403.69 ± 53.71 cm/s, *t* = 1.84, ES 2.60, Figure 3A) and MAP of the lower limb (93.07 ± 2.17 vs. 100.42 ± 3.59 mmHg, *t* = 1.75, ES 2.48, Figure 3D) were significantly lower in the HT group than in the LT group (*p* < 0.05). Nonetheless, the ABI (HT: 1.13 ± 0.02 vs. LT: 1.13 ± 0.01, *t* = 0.14, ES 0.20), arm MAP (HT: 92.24 ± 2.25 vs LT: 97.45 ± 3.01 mmHg, *t* = 1.39, ES 1.96) were no statistical difference between HT and LT groups (*p* > 0.05) (Figure 3B,C).

## 4. Discussion

This is the first study that compares the tennis-playing time in the metabolic and cardiovascular health state in middle-lifespan individuals with comparable daily physical activity levels. The primary findings of this study are that, under the condition of equivalent daily physical activity level, the lower degree of arterial stiffness (lower PWV) and insulin resistance were found in the elderly with higher tennis-playing time (HT group) when compared to those with lower tennis-playing time (LT group). Our current results imply that the elderly-lifespan of individuals who spend more time involved in tennis-playing may exhibit greater arterial compliance (less arterial stiffness) and insulin sensitivity benefits.

After experimental recruitment, all participants were allocated into high tennis time (HT) and low tennis time (LT) groups using stratified analysis mode [28]. There was no significant difference in their basic and anthropometric measurements, and the years of tennis-playing were comparable between groups. However, consistent with the stratified analysis result (time of tennis) used in this study, the HT group displayed higher frequency (play tennis per week), daily playing tennis hours, weekly total playing tennis time than the LT group (Table 1). In particular, there was no difference in the physical activity time per week between the two groups (Figure 2). Therefore, when there is no difference between the basic anthropometry parameters, the difference in the amount of weekly tennis exercise is likely to cause differences in vascular function and insulin resistance.

Several lines of evidence reveal that tennis can improve cardiopulmonary function, reduce the risk for cardiovascular disease and stress, and develop a strong heart and better fitness levels [9,10,29], lower insulin concentration during exercise [30], and enhance the capacity to cope with stress [31,32]. Although the characteristics of tennis matches have variations among different player styles and court surfaces, the characteristics of a tennis match included: 30 min to several hours of match time, several kilometers running distance, 2 to 12 s of each point duration, 3-7METs intensity, and 63–87% average heart rate, therefore, the intensity of tennis could be classified as moderately vigorous [9,11,12]. One of the main features of tennis is the HIIT model. Intervention studies reveal that following a period of HIIT training can improve insulin sensitivity which is determined via HOMA-IR [33,34]. Moreover, the tennis-related health benefits described above may contribute to better cardiovascular function and metabolic biomarker profiles (e.g., insulin sensitivity) in tennis participants with long-term playing years. Insulin resistance has been recognized as the common origin of several degenerative diseases, including cardiovascular disease [35,36]. However, more related evidence is still needed in future studies.

Here we found that the HT showed lower in baPWV (1284 ± 37 vs. 1404 ± 54 cm/s, *p* < 0.05) and lower limb MAP (93 ± 2 vs. 100 ± 4 mmHg, *p* < 0.05) than LT (Figure 3). First of all, previous evidence revealed that those engaged in vigorous exercise will experience increased blood flow to the working muscles and produce a greater ability to extract oxygen into the tissue [37]. This peripheral adaptability is due to the ability of the skeletal muscle blood vessels vasodilation as needed during maximum exercise. Furthermore, the moving limbs cause arterial dilatation effects which can improve blood flow to the heart. Exercise-induced shear stress can also stimulate flow-mediated endothelial function, resulting in vascular remodeling [38,39,40,41]. Therefore, these factors explained the positive effects of tennis on vascular functions. Indeed, epidemiological study evidence indicated that people involved in long-term racquet exercise can reduce the all-cause and cardiovascular-disease mortality which was a positive correlation associated with the exercise time [13]. Furthermore, the nature of tennis in some way is similar to HIIT (moderately vigorous), and the cardiovascular benefits obtained by tennis participation in elderly populations might be explained by the beneficial effects of HIIT on cardiovascular function and structure remodeling [42,43,44]. However, it might be the potential mechanism we inferred, and more research evidence is needed to confirm in future studies. Besides, Ankle Brachial Index (ABI) is a simple, non-invasive diagnostic test for peripheral arterial disease (PAD). According to the American Heart Association (AHA) guidelines, the ABI ≤ 0.90 had been suggested to identify PAD with serious stenosis [45]. Previously study found that physical activity significantly associates with improved ABI values among healthy individuals with borderline ABI [46]. Furthermore, a randomized trial reported that the 6-month exercise program could significant improvement in ABI in uncomplicated type 2 diabetes individuals [47]. In our study results, there was no difference between the two groups, and we suggested both groups are considered to be the health group, and the result of regular exercise makes no difference in ABI between the two groups.

Subsequently, we assumed that the amount of tennis per week is another key factor that causes cardiovascular adaption. In the study results, the total physical activity level for both groups was equal (LT: 54.9 ± 5.1 per week, HT: 54.0 ± 6.1 per week), but the HT group participated in more tennis activity weekly (Table 1), Moreover, the LT group showed less participation in high-intensity activities in the weekly physical activity record. (Figure 2). Thus, the more time engaged in high-intensity physical activity per week might be an important factor in metabolic and cardiovascular health benefits gain in this study. The previous cross-sectional study indicated that elderly populations can have lower arterial stiffness when they have higher daily time spent in light physical activity [28]. However, this phenomenon had not been observed in young populations. Such results were also observed in this elderly tennis population’s research. It is worth noting that a previous longitudinal cohort study investigating the elderly population showed that adequate physical activity and participating in vigorous physical activity may reduce arterial stiffness [48]. Other studies also showed the association between more regular high-intensity exercise and lower levels of arteriosclerosis [49,50]. The results of our study are the same as the above research evidence. When compared the high and low tennis exercise time per week, even though the total physical activity is the same and there was no any significant difference in body composition (weight, fat%, and muscle mass) and chronic inflammatory degree (CRP), the higher time of tennis per week would have lower arterial stiffness. The blood lipid biomarkers were no differences between HT and LT (Table 2). We speculate that the overall health states might be similar between the middle-aged participants in this study. Although there were still existing differences in the level of tennis participation and duration of high-intensity activity, there were no significant differences in blood lipid profiles between groups. The reason could be due to the participants were regular in exercise and had normal activity levels. However, it is worth noting that insulin sensitivity was significantly higher in the high-tennis playing group than in the low-tennis playing group. Since insulin sensitivity is a more sensitive indicator of metabolic health in comparison with lipid profiles, our findings further highlight the potential benefits of increased tennis participation in improving metabolic health in the middle-aged and elderly population. Taken together, the racquet and HIIT nature of tennis, combined with the higher regular physical activity might cause a better cardiovascular response. The phenomenon was confirmed by our present results.

The beneficial effects of regular exercise on blood pressure and insulin resistance have been demonstrated [4,5,51]. There is a close relationship between blood pressure and vascular function because the endothelial function plays a major role in regulating peripheral vascular resistance and blood pressure [52]. Regular exercise, especially aerobic endurance exercise, can increase nitric oxide (NO) production which may have beneficial effects on the antihypertensive effect by endogenous NO [53]. In this study results, we found the lower MAP and HOMA-IR in the HT group than the LT group, thus such greater BP and insulin sensitivity might be attributed to the greater time of tennis-playing. We suggested that the higher tennis involved time per week might have beneficial effects on blood pressure and insulin sensitivity, and the possible mechanism included the increase of NO production, peripheral resistance decrease, maintain muscle mass during aging. Moreover, the previous study indicated that an increase in weekly exercise frequency has additional benefits for the vitality of the elderly [54]. The previous findings were in line with the potential benefits of arterial stiffness parameters observed in this study. However, more relevant evidence is needed to explain the relationship between blood pressure and insulin sensitivity. Moreover, although there was no significant difference in the years of tennis participation between subjects in the low- and high-tennis exercise time groups, we found that the average years of tennis participation period in the high-tennis time group was approximately ~6 years longer than in the low-tennis time group. Therefore, we may not be able to rule out a possible influence of the difference in years of tennis participation on the changes in cardiometabolic health. However, there are still lack of longitudinal evidence directly comparing the variations of the prolonged periods of exercise participation on cardiometabolic health benefits. Future interventional studies are encouraged to focus on the relationships between tennis participation years and cardiovascular health benefits to better elucidate and clarify possible effects and mechanisms.

The other considerable limitations of this study should be mentioned here. Firstly, the questionnaire is used in this study to investigate the physical activity, rather than direct measurements. Although many previous studies have adopted questionnaires to investigate the amount of physical activity, the lack of actual measurement of physical activity or the fitness status of the subjects (i.e., VO_2_max) is still one of the limitations of this study. Secondly, due to the specific of the subject population needed (age, exercise habits, tennis ball age, health status, lifestyle habits, and willingness to participate); the subject sample size could be one of the limitations of this study. It is suggested that follow-up studies can include more tennis participants of different age groups. Thirdly, we use a cross-sectional study to explore the mechanism of tennis training time on the above indicators, however, interventional research is also recommended for further investigation. Fourthly, the LT group played about 4.5 h of tennis per week. With a frequency of 1.5 h of exercise three times a week, the LT group is considered to meet the recommended amount of exercise prescription [55]. The “low time” used in LT group of this study are only the results from stratification analysis and are different from the general population. Another limitation of this study was that a recall survey approach and was unable to determine a causal relationship between the time of tennis participation and cardiometabolic health biomarkers. Therefore, future studies employing interventional or longitudinal designs are warrant to clarify the causal relationship between tennis exercise time and cardiometabolic health benefits. Finally, some relevant biological indicators (e.g., circulating nitric oxide level) were not directly measured in this study, thus another limitation of this study to include relevant potential mechanisms for the benefits of tennis participation in the discussion. Through the evidence of this study, for elderly tennis participants, it can provide the relationship between the amount of tennis participation and atherosclerosis, metabolic biomarkers, and insulin sensitivity.

## 5. Conclusions

In conclusion, we demonstrated that under the condition of similar daily physical activity levels, the elderly with a higher time of tennis-playing (HT group) exhibited relatively lower arterial stiffness (lower PWV) and lower insulin resistance when compared to those with lower time tennis-playing (LT). Our current findings imply that even though under the comparable time of daily physical activity, the lifespan of elderly individuals who spend more time involved in tennis-playing may exhibit greater benefits on cardiometabolic health (i.e., greater arterial compliance and insulin sensitivity). Moreover, our findings raise the feasibility of constructing more appropriate racquet sport facilities/environments (such as easy access to tennis courts) in promoting public health in the populations with advancing aging.

## Figures and Tables

**Table 1:** Physical characteristics of participants.

Variables\Group	HT (*n* = 13, 8 M/5 F)	LT (*n* = 13, 7 M/6 F)	*t*
Height (cm)	166.04 ± 1.83	162.21 ± 2.00	−1.41
Wight (kg)	63.30 ± 2.59	62.90 ± 2.90	−0.10
BMI (kg/m^2^)	22.92 ± 0.78	23.42 ± 0.61	0.51
Age (year)	61.54 ± 1.32	60.89 ± 1.07	−0.38
Tennis participated years (year)	28.62 ± 0.84	22.77 ± 3.52	−1.62
Tennis play frequency (days/week)	5.46 ± 0.24	3.27 ± 0.41	−4.66 **
Tennis play hours (h/day)	2.60 ± 0.22	1.33 ± 0.14	−4.92 **
Total tennis play time (h/week)	14.02 ± 1.32	4.47 ± 0.67	−6.45 **

** indicating a statistically significant difference between the two groups *p* < 0.05.

**Table 2:** Differences in serum marker levels according to the amount of tennis played.

Variables\Group	HT *n* = 13	LT *n* = 13	*t*	*ES*
Glucose (mg/dL)	92.46 ± 1.29	95.38 ± 1.98	1.24	0.48
CHOL (mg/dL)	215.69 ± 8.37	208.92 ± 7.40	−0.61	0.24
TG (mg/dL)	86.77 ± 12.16	65.46 ± 7.03	−1.52	0.60
HDL (mg/dL)	66.56 ± 4.67	62.94 ± 4.01	−0.59	0.23
LDL (mg/dL)	126.08 ± 8.56	124.46 ± 5.14	−0.16	0.06
CRP (mg/dL)	0.40 ± 0.23	0.09 ± 0.03	−1.32	0.05
Insulin (μU/mL)	6.17 ± 0.49	9.55 ± 1.92	1.70 *	0.67
HOMA-IR	1.41 ± 0.11	2.27 ± 0.48	1.76 *	0.69

* indicating a statistically significant difference between the two groups (*p* < 0.05).

## Data Availability

The data presented in this study are available upon request from the corresponding author.

## References

[1] Sui X., LaMonte M.J., Laditka J.N., Hardin J.W., Chase N., Hooker S.P., Blair S.N. (2007). Cardiorespiratory fitness and adiposity as mortality predictors in older adults. J. Am. Med. Assoc..

[2] Powell K.E., Thompson P.D., Caspersen C.J., Kendrick J.S. (1987). Physical activity and the incidence of coronary heart disease. Annu. Rev. Public Health.

[3] Blair S.N., Kohl H.W., Paffenbarger R.S., Clark D.G., Cooper K.H., Gibbons L.W. (1989). Physical fitness and all-cause mortality: A prospective study of healthy men and women. J. Am. Med. Assoc..

[4] Shephard R.J., Balady G.J. (1999). Exercise as cardiovascular therapy. Circulation.

[5] Ryan A.S. (2000). Insulin resistance with aging: Effects of diet and exercise. Sports Med..

[6] Deiseroth A., Streese L., Köchli S., Wüst R.S., Infanger D., Schmidt-Trucksäss A., Hanssen H. (2019). Exercise and arterial stiffness in the elderly: A combined cross-sectional and randomized controlled trial (examin age). Front. Physiol..

[7] Hussain S.R., Macaluso A., Pearson S.J. (2016). High-intensity interval training versus moderate-intensity continuous training in the prevention/management of cardiovascular disease. Cardiol. Rev..

[8] Groppel J., DiNubile N. (2009). Tennis: For the health of it!. Phys. Sportsmed..

[9] Kovacs M.S. (2007). Tennis physiology: Training the competitive athlete. Sports Med..

[10] Marks B. (2006). Health benefits for veteran (senior) tennis players. Br. J. Sports Med..

[11] Fernandez-Fernandez J., Sanz-Rivas D., Sanchez-Muñoz C., Pluim B.M., Tiemessen I., Mendez-Villanueva A. (2009). A comparison of the activity profile and physiological demands between advanced and recreational veteran tennis players. J. Strength Cond. Res..

[12] Bekraoui N., Fargeas-Gluck M.A., Léger L. (2012). Oxygen uptake and heart rate response of 6 standardized tennis drills. Appl. Physiol. Nutr. Metab..

[13] Oja P., Kelly P., Pedisic Z., Titze S., Bauman A., Foster C., Hamer M., Hillsdon M., Stamatakis E. (2016). Associations of specific types of sports and exercise with all-cause and cardiovascular-disease mortality: A cohort study of 80306 British adults. Br. J. Sports Med..

[14] Chomistek A.K., Cook N.R., Flint A.J., Rimm E.B. (2012). Vigorous-intensity leisure-time physical activity and risk of major chronic disease in men. Med. Sci. Sports Exerc..

[15] Hasegawa N., Fujie S., Horii N., Miyamoto-Mikami E., Tsuji K., Uchida M., Hamaoka T., Tabata I., Iemitsu M. (2018). Effects of different exercise modes on arterial stiffness and nitric oxide synthesis. Med. Sci. Sports Exerc..

[16] Su L., Fu J., Sun S., Zhao G., Cheng W., Dou C., Quan M. (2019). Effects of HIIT and MICT on cardiovascular risk factors in adults with overweight and/or obesity: A meta-analysis. PLoS ONE.

[17] Jelleyman C., Yates T., O’Donovan G., Gray L.J., King J.A., Khunti K., Davies M.J. (2015). The effects of high-intensity interval training on glucose regulation and insulin resistance: A meta-analysis. Obes. Rev..

[18] Hemmatinafar M., Kordi M., Choopani S., Choobineh S., Gharari Arefi R. (2013). The effect of high intensity interval training (HIIT) on plasma adiponectin levels, insulin sensitivity and resistance in sedentary young men. J. Adv. Med. Biomed. Res..

[19] Hwang C.L., Yoo J.K., Kim H.K., Hwang M.H., Handberg E.M., Petersen J.W., Christou D.D. (2016). Novel all-extremity high-intensity interval training improves aerobic fitness, cardiac function and insulin resistance in healthy older adults. Exp. Gerontol..

[20] Lakatta E.G. (2003). Arterial and cardiac aging: Major shareholders in cardiovascular disease enterprises part III: Cellular and molecular clues to heart and arterial aging. Circulation.

[21] Mattace-Raso F.U.S., van der Cammen T.J.M., Hofman A., van Popele N.M., Bos M.L., Schalekamp M.A.D.H., Asmar R., Reneman R.S., Hoeks A.P.G., Breteler M.M.B. (2006). Arterial stiffness and risk of coronary heart disease and stroke. Circulation.

[22] Garber C.E., Blissmer B., Deschenes M.R., Franklin B.A., Lamonte M.J., Lee I.M., Nieman D.C., Swain D.P. (2011). Quantity and quality of exercise for developing and maintaining cardiorespiratory, musculoskeletal, and neuromotor fitness in apparently healthy adults: Guidance for prescribing exercise. Med. Sci. Sports Exerc..

[23] Muniyappa R., Sowers J.R. (2013). Role of insulin resistance in endothelial dysfunction. Rev. Endocr. Metab. Disord..

[24] Jia G., Sowers J.R. (2014). Endothelial dysfunction potentially interacts with impaired glucose metabolism to increase cardiovascular risk. Hypertension.

[25] Bouchard C., Tremblay A., Leblanc C., Lortie G., Savard R., Thériault G. (1983). A method to assess energy expenditure in children and adults. Am. J. Clin. Nutr..

[26] Yamashina A., Tomiyama H., Takeda K., Tsuda H., Arai T., Hirose K., Koji Y., Hori S., Yamamoto Y. (2002). Validity, reproducibility, and clinical significance of noninvasive brachial-ankle pulse wave velocity measurement. Hypertens. Res..

[27] Matthews D.R., Hosker J.P., Rudenski A.S., Naylor B.A., Treacher D.F., Turner R.C. (1985). Homeostasis model assessment: Insulin resistance and beta-cell function from fasting plasma glucose and insulin concentrations in man. Diabetologia.

[28] Gando Y., Yamamoto K., Murakami H., Ohmori Y., Kawakami R., Sanada K., Higuchi M., Tabata I., Miyachi M. (2010). Longer time spent in light physical activity is associated with reduced arterial stiffness in older adults. Hypertension.

[29] Davis M.G., Fox K.R. (2007). Physical activity patterns assessed by accelerometry in older people. Eur. J. Appl. Physiol..

[30] Ferrauti A., Bergeron M.F., Pluim B.M., Weber K. (2001). Physiological responses in tennis and running with similar oxygen uptake. Eur. J. Appl. Physiol..

[31] Pluim B.M., Groppel J.L., Miley D., Crespo M., Turner M.S. (2018). Health benefits of tennis. Br. J. Sports Med..

[32] Kovacs M., Pluim B., Groppel J., Crespo M., Roetert E.P., Hainline B., Miller S., Reid M., Pestre B., De Vylder M. (2016). Health, Wellness and Cognitive Performance Benefits of Tennis. J. Med. Sci. Tennis.

[33] Mitranun W., Deerochanawong C., Tanaka H., Suksom D. (2014). Continuous vs interval training on glycemic control and macro- and microvascular reactivity in type 2 diabetic patients. Scand. J. Med. Sci. Sports.

[34] Marcinko K., Sikkema S.R., Samaan M.C., Kemp B.E., Fullerton M.D., Steinberg G.R. (2015). High intensity interval training improves liver and adipose tissue insulin sensitivity. Mol. Metab..

[35] Krentz A.J., Viljoen A., Sinclair A. (2013). Insulin resistance: A risk marker for disease and disability in the older person. Diabet. Med..

[36] Ormazabal V., Nair S., Elfeky O., Aguayo C., Salomon C., Zuñiga F.A. (2018). Association between insulin resistance and the development of cardiovascular disease. Cardiovasc. Diabetol..

[37] Joyner M.J., Casey D.P. (2015). Regulation of increased blood flow (hyperemia) to muscles during exercise: A hierarchy of competing physiological needs. Physiol. Rev..

[38] Tinken T.M., Thijssen D.H., Hopkins N., Dawson E.A., Cable N.T., Green D.J. (2010). Shear stress mediates endothelial adaptations to exercise training in humans. Hypertension.

[39] Green D.J., Fowler D.T., O’Driscoll J.G., Blanksby B.A., Taylor R.R. (1996). Endothelium-derived nitric oxide activity in forearm vessels of tennis players. J. Appl. Physiol..

[40] Kagaya A., Ohmori F., Okuyama S., Muraoka Y., Sato K. (2010). Blood flow and arterial vessel diameter change during graded handgrip exercise in dominant and non-dominant forearms of tennis players. Adv. Exp. Med. Biol..

[41] Green D.J., Smith K.J. (2018). Effects of exercise on vascular function, structure, and health in humans. Cold Spring Harb. Perspect. Med..

[42] Ramos J.S., Dalleck L.C., Tjonna A.E., Beetham K.S., Coombes J.S. (2015). The impact of high-intensity interval training versus moderate-intensity continuous training on vascular function: A systematic review and meta-analysis. Sports Med..

[43] Ashor A.W., Lara J., Siervo M., Celis-Morales C., Mathers J.C. (2014). Effects of Exercise Modalities on Arterial Stiffness and Wave Reflection: A Systematic Review and Meta-Analysis of Randomized Controlled Trials. PLoS ONE.

[44] Tordi N., Mourot L., Colin E., Regnard J. (2010). Intermittent versus constant aerobic exercise: Effects on arterial stiffness. Eur. J. Appl. Physiol..

[45] Aboyans V., Criqui M.H., Abraham P., Allison M.A., Creager M.A., Diehm C., Fowkes F.G.R., Hiatt W.R., Jönsson B., Lacroix P. (2012). Measurement and interpretation of the ankle-brachial index: A scientific statement from the American Heart Association. Circulation.

[46] Heikkilä A., Venermo M., Kautiainen H., Aarnio P., Korhonen P. (2016). Physical Activity Improves Borderline Ankle–Brachial Index Values in a Cardiovascular Risk Population. Ann. Vasc. Surg..

[47] Gibbs B.B., Dobrosielski D.A., Althouse A.D., Stewart K.J. (2013). The effect of exercise training on ankle-brachial index in type 2 diabetes. Atherosclerosis.

[48] Endes S., Schaffner E., Caviezel S., Dratva J., Autenrieth C.S., Wanner M., Martin B., Stolz D., Pons M., Turk A. (2016). Long-term physical activity is associated with reduced arterial stiffness in older adults: Longitudinal results of the SAPALDIA cohort study. Age Ageing.

[49] Sugawara J., Otsuki T., Tanabe T., Hayashi K., Maeda S., Matsuda M. (2006). Physical activity duration, intensity, and arterial stiffening in postmenopausal women. Am. J. Hypertens..

[50] Van de Laar R.J., Ferreira I., van Mechelen W., Prins M.H., Twisk J.W., Stehouwer C.D. (2010). Lifetime vigorous but not light-to-moderate habitual physical activity impacts favorably on carotid stiffness in young adults: The Amsterdam growth and health longitudinal study. Hypertension.

[51] Way K.L., Hackett D.A., Baker M.K., Johnson N.A. (2016). The effect of regular exercise on insulin sensitivity in type 2 diabetes mellitus: A systematic review and meta-analysis. Diabetes Metab..

[52] Sandoo A., van Zanten J.J.V., Metsios G.S., Carroll D., Kitas G.D. (2010). The endothelium and its role in regulating vascular tone. Open Cardiovasc. Med. J..

[53] Maeda S., Tanabe T., Otsuki T., Sugawara J., Iemitsu M., Miyauchi T., Kuno S., Ajisaka R., Matsuda M. (2004). Moderate regular exercise increases basal production of nitric oxide in elderly women. Hypertens. Res..

[54] Rugbeer N., Ramklass S., Mckune A., Van Heerden J. (2017). The effect of group exercise frequency on health related quality of life in institutionalized elderly. Pan Afr. Med. J..

[55] Haskell W.L., Lee I.M., Pate R.R., Powell K.E., Blair S.N., Franklin B.A., Macera C.A., Heath G.W., Thompson P.D., Bauman A. (2007). Physical activity and public health: Updated recommendation for adults from the American College of Sports Medicine and the American Heart Association. Circulation.

